# In-Depth Analysis of the Plasma Proteome in ME/CFS Exposes Disrupted Ephrin-Eph and Immune System Signaling

**DOI:** 10.3390/proteomes9010006

**Published:** 2021-01-29

**Authors:** Arnaud Germain, Susan M. Levine, Maureen R. Hanson

**Affiliations:** Department of Molecular Biology and Genetics, Cornell University, Ithaca, NY 14853, USA; ag297@cornell.edu (A.G.); cfssuelev@earthlink.net (S.M.L.)

**Keywords:** ME/CFS, proteomics, plasma, ephrin-Eph pathway, immune metabolism, adherens junction, glucose, SOMAscan^®^, diagnosis

## Abstract

Myalgic encephalomyelitis/chronic fatigue syndrome (ME/CFS) is a disabling disease with worldwide prevalence and limited therapies exclusively aimed at treating symptoms. To gain insights into the molecular disruptions in ME/CFS, we utilized an aptamer-based technology that quantified 4790 unique human proteins, allowing us to obtain the largest proteomics dataset yet available for this disease, detecting highly abundant proteins as well as rare proteins over a nine-log dynamic range. We report a pilot study of 20 ME/CFS patients and 20 controls, all females. Significant differences in the levels of 19 proteins between cohorts implicate pathways related to the extracellular matrix, the immune system and cell–cell communication. Outputs of pathway and cluster analyses robustly highlight the ephrin pathway, which is involved in cell–cell signaling and regulation of an expansive variety of biological processes, including axon guidance, angiogenesis, epithelial cell migration, and immune response. Receiver Operating Characteristic (ROC) curve analyses distinguish the plasma proteomes of ME/CFS patients from controls with a high degree of accuracy (Area Under the Curve (AUC) > 0.85), and even higher when using protein ratios (AUC up to 0.95), that include some protein pairs with established biological relevance. Our results illustrate the promise of plasma proteomics for diagnosing and deciphering the molecular basis of ME/CFS.

## 1. Introduction

The illness variously known as myalgic encephalomyelitis or chronic fatigue syndrome (ME/CFS) profoundly affects the quality of life of its victims. Predominant symptoms are not only exhaustion, but also malaise, pain, orthostatic intolerance, brain fog, and exacerbation of symptoms following mental or physical activity [1,2]. Even though ME/CFS patients exhibit a wide range of symptoms, standard blood tests ordered by physicians usually have values in normal ranges, leading to misdiagnoses of depression or other psychiatric illness by individuals not familiar with the disease. Objective biomarkers are therefore urgently needed to facilitate diagnosis, apply appropriate treatments, and avoid counterproductive recommendations, by monitoring the efficacy of experimental drugs or other therapies, as well as provide information that might reveal underlying causes of the disease.

A number of molecules have been measured in plasma or serum in attempts to identify biomarkers. Cytokines were among the first to be monitored, but metabolomic and proteomic studies have followed as technology has improved [3,4,5,6,7,8,9,10,11]. Likewise, a number of abnormalities have been observed in immune cells, ranging from altered energy metabolism to reduced activity [12,13,14,15,16,17]. While these assays demonstrate that the illness has a biological basis, they are usually more suited for a research laboratory than for a diagnostic service. Being able to identify the illness with a small number of biomarkers present in blood or other readily obtainable bodily fluids would facilitate the development of a widely available test.

We decided to carry out a pilot study on a carefully selected small population of ME/CFS cases and controls to take advantage of an aptamer-based proteomic assay to measure an unusually large number of plasma proteins. Our encouraging results indicate that this approach is a promising method to identify biomarkers that could aid in diagnosis. More importantly, it unveils key aspects of biology that are disrupted in patients suffering from ME/CFS.

## 2. Materials and Methods

### 2.1. Cohort and Blood Sampling

The patient cohort selected for this study consists of 20 females diagnosed by Susan Levine (M.D.), an ME/CFS specialist located in New York City (NYC). All patients fulfilled the Fukuda criteria [18]. She selected 20 healthy controls with similar age and BMI from the same location.

The blood collection protocol was identical to the one described in Germain et al. (2020) [19] and the same survey data was collected and compiled to evaluate the clinical status of both cohorts.

### 2.2. Proteomics Data Acquisition and Handling

Plasma, thawed once for aliquoting purposes, was shipped on dry ice to SomaLogic, Inc. in Boulder, CO (www.somalogic.com), where the samples’ relative protein abundance was measured using the SOMAscan Assay. We were provided with data that was expressed in continuous relative fluorescence units, which are derived from the hybridization of Slow Offrate Modified Aptamers (SOMAmer^®^) reagents to a custom DNA microarray after they were exposed to our individual plasma samples.

There were two datasets that had different levels of standardization performed by SomaLogic. One of them included all the steps and is the dataset used throughout most of this manuscript. In brief, normalization steps are used to remove hybridization variation within a run, biases within a run, intensity difference between runs, assay difference between runs, and finally references are used for the final normalization. The second one had a somewhat fewer normalization steps applied and Table S2 is the only analysis derived from the second dataset.

Aptamers were available to measure 5284 protein abundances, of which, 4979 are for detection of human proteins, another 252 belong to 13 distinct organisms spanning the tree of life, and 53 were used as varying controls and references. The aptamers were designed to detect 4790 unique proteins, with 203 targeted to two or more parts of a same protein. The experiment was set up on two separate plates with the required buffer, calibrator and quality control (QC) wells. Each plasma sample was split into three dilutions to account for natural human plasma protein abundance, 4022 proteins were measured at 20% dilution, 797 at 0.5% dilution, and 160 at 0.005% dilution. Finally, a total of 248 proteins were “flagged” based on SomaLogic’s acceptance criteria. Those proteins, when they were significantly different between cohorts, were nevertheless included in our final report, as this is an exploratory study and including them results in a marginal decrease of the significance for our statistical tests.

### 2.3. Data Analysis

A nonparametric Wilcoxon rank-sum test was chosen to test for significant differences between each cohort, since numerous variables failed the Shapiro test of normality. The false discovery rate (FDR) was controlled for multiple testing by using the Benjamini–Hochberg (BH) correction method and reported as *q*-values.

Analyses solely based on fold-change as well as a volcano plot analysis failed due to the presence of severe outliers for a few variables. As those outliers were not consistent with a specific subject, it is not possible to eliminate them and while the Wilcoxon rank-sum test is able to circumvent such values due to its design, any analysis solely relying on fold-change data input was not included in our analysis. Nevertheless, fold changes are included in our results to illustrate both the range and the direction of the differences for the proteins discussed.

Protein functional interactions were assessed using the online tool and database, STRING 11.0 (https://string-db.org). Both the “Multiple proteins” and the new “Proteins with Values/Ranks” menus were used to query the database and the enrichment of our dataset for certain pathways in *Homo sapiens*.

The receiver operating characteristic (ROC) curves were obtained through the independent statistical module (Biomarker Analysis) provided by MetaboAnalyst 5.0 (www.metaboanalyst.ca. No filtering was performed, the 100 top ratios were computed and included. Finally, the data was log-transformed and auto-scaled before the ROC curves were generated.

Functional annotation clustering was investigated using the Database for Annotation, Visualization and Integrated Discovery (DAVID) v6.8 (https://david.ncifcrf.gov).

Correlation calculations were performed using R.

## 3. Results

### 3.1. Population Statistics

The population described in Table 1 is exclusively female, equally divided between controls and patients (20 each). As displayed, both cohorts were similar for age and BMI (*t*-test *p* = 0.43 and 0.57, respectively). Additional elements displayed in Table 1 depict relevant symptoms endured by the ME/CFS cohort as well as their intensity based on two distinct but complementary scales, namely the Bell scale and the more detailed 36-Item Short Form Survey (SF-36). Both SF-36 metrics are significantly different between cohorts with a *t*-test *p* < 0.0001. The observed gap between cohorts undoubtedly represents the burden of illness in the patient community.

### 3.2. Nine Proteins Are Significantly Higher in ME/CFS Patients

**Figure 1:**
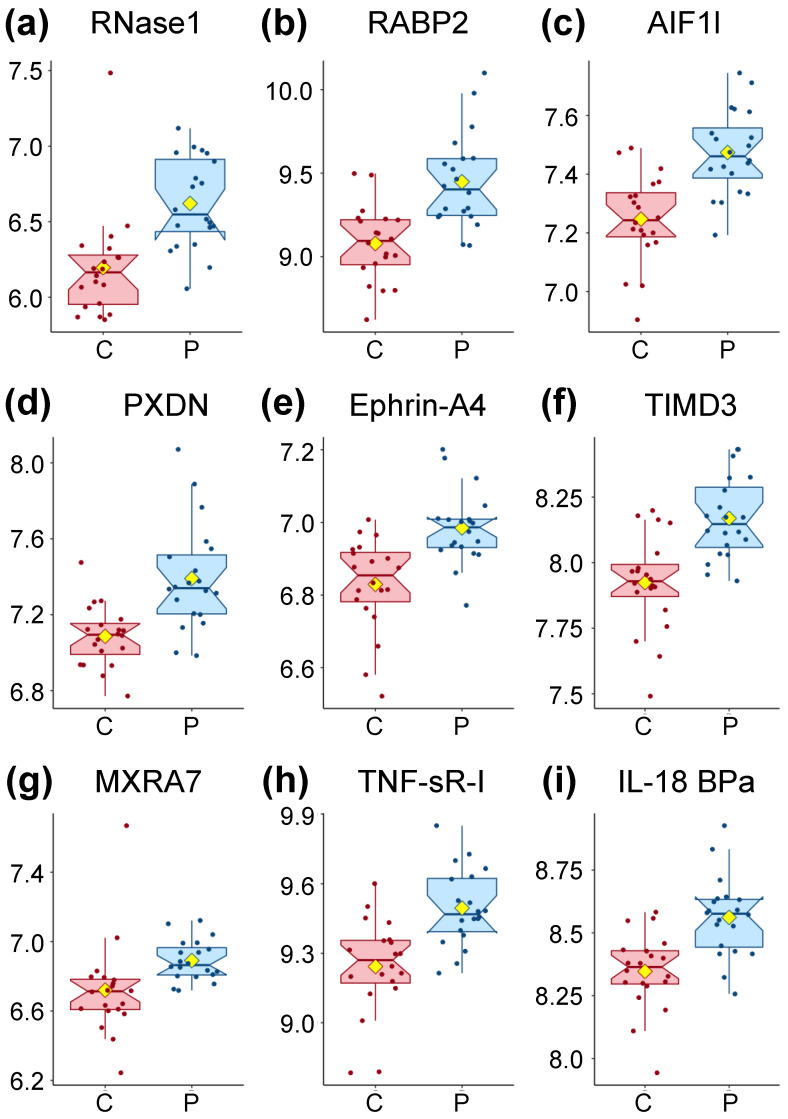
Box plot distribution of logged values for the proteins significantly different between controls and patients through Wilcoxon rank-sum testing with *p* < 0.05 and *q* < 0.05 displayed in Table 2. Controls (C) are shown in red and Myalgic Encephalomyelitis/Chronic Fatigue Syndrome (ME/CFS) patients (P) in blue. The yellow diamond represents the mean.

Out of the 4979 aptamers used, 391 proteins were found significantly different at a threshold of *p* < 0.05 (Supplementary File 1) and nine were significantly different at a threshold of both *p* < 0.05 and *q* < 0.05. Of the latter, all are higher in patients compared to controls (Figure 1 and Table 2). We consulted several databases (www.uniprot.org, https://ibioguide.advaitabio.com, https://string-db.org) that describe human plasma proteins in order to determine what is currently known about the proteins found to have accumulated at different levels.

RNase1 (Figure 1a) is a secreted endoribonuclease and favors the cleavage of double-stranded over single-stranded RNA. RNase 1 forms a tight 1:1 complex with RNH1, resulting in its inhibition but RNH1 is not part of our dataset.

RABP2 (Figure 1b) is a cytosol-to-nuclear shuttling protein, facilitating access of retinoic acid (vitamin A) to the nuclear receptors. This protein is also associated with increased circulating low-density lipoprotein cholesterol (LDL-C).

AIF1L (Figure 1c) is described only as an actin-binding protein promoting actin bundling.

PXDN (Figure 1d) is secreted into the extracellular matrix where it is involved in its formation. It also participates in H_2_O_2_ metabolism and peroxidative reactions in the cardiovascular system. Ca^2+^ and heme b are both cofactors of PXDN. PXDN is induced by TGFB1 in fibroblasts and upregulated in apoptotic cells. TGFB1 was found slightly lower in patients for our population (FC = 0.97, Supplementary File 1). PXDN also blocks the binding of interleukin-1 (not measured in our dataset) to its receptor complex.

Ephrin-A4 (Figure 1e) is a member of the ephrin family, a ligand ephrin receptor protein-tyrosine kinase crucial for migration, repulsion, and adhesion during neuronal, vascular, and epithelial development. This is achieved by the creation of contact-dependent bidirectional signaling between adjacent cells, a mechanism also involved in immune cell sensing.

TIMD3 (Figure 1f) is a receptor implicated in modulating innate and adaptive immune responses, promoting immunological tolerance. Among many functions, TIMD3 regulates macrophage activation, inhibits-helper type 1 lymphocytes (Th1), attenuates TCR-induced signaling in CD8+ cells, and suppresses NK cell-mediated cytotoxicity.

MXRA7 (Figure 1g) has been found to be mostly expressed in ocular tissues with some evidence for its role in neovascularization and healing.

TNF SR-I (Figure 1h) is a membrane-bound receptor for tumor necrosis factor alpha (TNF-α) as well as a soluble receptor when proteolytically processed, and plays a role in cell survival, apoptosis and inflammation.

Finally, IL-18 BPa (Figure 1i) inhibits IL-18 activity by binding to it, inhibiting the early Th1 cytokine response which tends to produce proinflammatory responses. Elevated levels of this protein are detected in the intestinal tissues of patients with Crohn’s disease.

To summarize, two proteins (PXDN and MXRA7) are involved in the extracellular matrix, while AIF1L plays a role in the cytoskeleton. Four of the proteins (Ephrin-A4, TIMD3, TNF SR-I, and IL-18 BPa) can be linked to the immune response, with TIMD3 and IL-18 BPa having an inhibitory role, while only the soluble form, but not the membrane-bound form, of TNF sR-I has an inhibitory role.

### 3.3. Additional Proteins Emerging from a Relaxed q-Value Threshold

If we relax the multiple testing threshold to *q* < 0.15, an additional eight proteins are classified as significantly different between ME/CFS patients and controls (Table S1 and Figure S1). All of these proteins exhibit *p*-values lower than 0.05.

Only one of them, CFTR, is lower in patients compared to controls (Figure S1g). CFTR is a chloride and bicarbonate ion channel located mainly in epithelial tissues, where it controls ion and water exchanges. Homozygous mutations in this gene cause cystic fibrosis. ROR1 is a cell surface receptor modulating neurite growth in the central nervous system, associated with B-cell chronic lymphocytic leukemia in case of increased expression. Both DCP1A and BAMBI are involved in the TGF-beta signaling pathway, while PPIC assists protein folding.

When we perform the same statistical analysis on the less standardized dataset provided, we have to increase the threshold to *q* < 0.2 to find four proteins significantly different between ME/CFS patients and controls (Table S2). Once again, all four are higher in patients compared to controls, with PPIC and ROR1 already present in Table S1 and Figure S1. DR6 is in the same family as TNF sR-I and is required for both normal cell body death and axonal pruning while it negatively regulates T-cell responses and cytokine release. Ephrin-A5 is in the same family as Ephrin-A4 (Table 2) and has been shown to be involved in various biological processes including cell–cell adhesion, cytoskeletal organization, axon fasciculation, brain development, and glucose-stimulated insulin secretion through pancreatic islet cell communications.

### 3.4. Protein–Protein Association Analysis Points Both to the Ephrin Family and Immune Metabolism

We used the STRING tool and its extensive database to identify changes in functional interactions of proteins between both cohorts.

**Figure 2:**
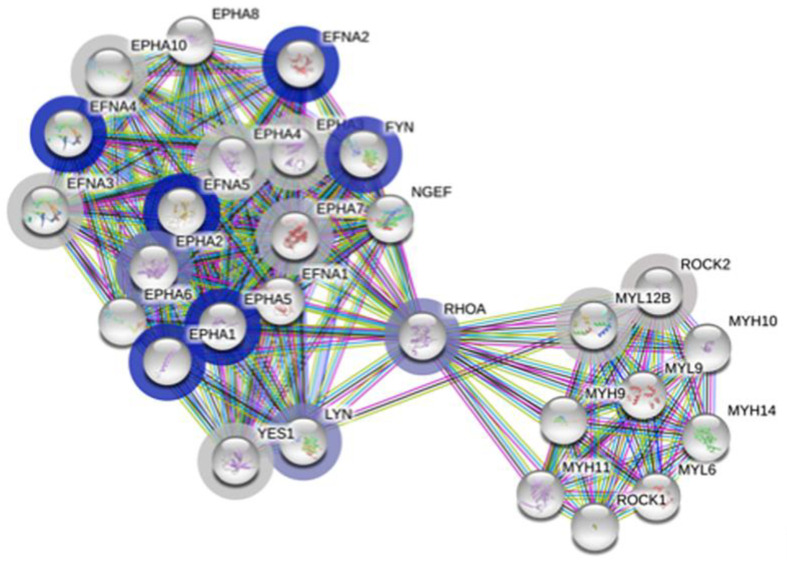
Graphical display of the EPHA-mediated growth cone collapse network (HSA-3928663) from https://string-db.org. Each bubble represents a node, with filled nodes having some known or predicted 3D structure. The halo color is based on the rank of the protein in the sorted set of input, log *p*-values in our case, with grey being less significant and darker blue being increasingly significant between controls and ME/CFS patients. Edges represent protein–protein interactions; known from curated databases (turquoise), known experimentally (purple), predicted from gene neighborhood (dark green), predicted from gene fusions (red), predicted from gene co-occurrence (dark blue); from text mining (light green), from coexpression (black) and from protein homology (light blue).

Our first query included the complete dataset along with the respective log *p*-value for each protein. The output of this query is based on rank testing and detects statistically enriched distribution of values in large lists of proteins. The functional enrichment analysis output outlined in Table 3 is clearly restricted to the proteins belonging to the family of ephrin receptors and ligands. The enrichment score is relatively high and the false discovery rate below 0.05. The largest pathway described, also inclusive of all others, is attached to the term “EPHA-mediated growth cone collapse” (Figure 2), with 28 proteins, of which 17 were measured in our dataset. Among those, the proteins with the lowest *p*-values (Table S3 and Figure S2) include four Ephrin type-A receptors and three Ephrin ligands, with EFNA4 and EFNA5 already mentioned in Table 2 and Table S2, respectively. The abundant interactions between these proteins and a few others are displayed in Figure 2 and include known and predicted interactions as well as others such as text mining, coexpression, and protein homology. Ephrin receptors are the largest known family of receptor tyrosine kinases (RTK) and mediate innumerable and essential processes in humans from embryonic development to adult tissue homeostasis through interactions with membrane-bound ephrin ligands. As both the receptors and the ligands are membrane-bound, cell–cell interaction is required for signaling to occur.

Three other proteins, namely, FYN, RhoA and LYN interact with all the proteins from the Ephrin family and all play an important role in countless aspects of cell–cell communication (Figure 2). Noteworthy is the fold change of these three proteins, which is inverted compared to the Ephrin family proteins, and is significantly higher in ME/CFS patients compared to controls (*p* < 0.15, Table S3 and Figure S2).

Our second query was limited to the 391 proteins with a *p* < 0.05 (Supplementary File 1) and therefore relied on STRING’s normal gene set-based analysis instead of the initial rank-based analysis. Here the input was the UniProt ID for each of the 391 proteins. As the output is lengthy and the results are easily repeatable using Supplementary File 1, the results mentioned below are not illustrated.

Many of the pathway descriptions from the varied functional enrichments of the network generated using this limited dataset still include the Ephrin signaling pathway. Other highly abundant pathways are centered around cell-to-cell communication and more particularly for regulation and activation of the different cell types of the immune system. If we focus on the Reactome Pathways as we did for the initial query, the pathway descriptions include “Immune system”, “Cytokine signaling in immune system”, “Platelet activation, signaling and aggregation”, “Platelet degranulation”, “Neutrophil degranulation”, and “Innate immune system” to only name a few. Other recurring aspects also centered around cell–cell communication are linked to cell adhesion, with adherens junctions, axon guidance and extracellular matrix organization.

### 3.5. High Levels of Prediction Are Achieved Using Univariate ROC Curve Analysis

A receiver operating characteristic (ROC) curve analysis was performed on the whole dataset to assess the ability to differentiate controls from ME/CFS patients. The areas under the curve (AUC) is used as a summary to represent the ability of a variable, in our case the abundance of a protein, to distinguish a healthy subject from a patient suffering from ME/CFS. AUCs are reported in Table 4 and Supplementary File 2, with higher AUCs indicating a better performance of the model to distinguish between the two groups.

Initially, we considered only proteins on their own (left two columns of Table 4). The top nine AUC values, higher than 0.85, were the same proteins as the ones significantly different between controls and patients (Table 2). Such a level of distinction between controls and patients is clearly illustrated in Figure 1, where the values of only a few subjects are overlapping with the box plot area (interquartile range—IQR) of the opposite cohort. Additional AUC values for individual proteins are reported in Supplementary File 2, starting at line 110.

We attained higher AUC values when protein ratios are computed for each protein pair, regardless of biological relevance, and for each subject before running the same type of analysis with this newly expanded dataset. The highest observed AUC was almost at 0.95 for the protein pair FXN-HAVCR2 and 97 pairs had a higher AUC then RNase1 and RABP2; the details for the first nine are displayed in the four columns to the right of Table 4, while the remaining ones can be found in Supplementary File 2.

The genes encoding FXN and HAVCR2 were both found to be differentially expressed during the immune response of mice CD4^+^ T-cells [20]. While HAVCR2 was mentioned in Table 2, Fraxatin (FDRA, FXN) is a mitochondrial protein with an in vitro ferroxidase activity which promotes the biosynthesis of heme as well as the assembly and repair of proteins containing iron-sulfur clusters.

Considering the second pair, Layilin (LAYN) is a receptor for hyaluronate and is a critical gene regulating T-cell function. CLEC4D, is the C-type lectin domain family 4 member D protein which drives the maturation of antigen-presenting cells and shapes antigen-specific priming of T-cell toward effector T-helper 1 and T-helper 17 cell subtypes. Therefore, it seems that the expression ratio of this pair of proteins has biological sense.

On the other hand, the RNASE1-ETNK1 pair does not seem to be relevant biologically. Indeed, RNase1 is a ribonuclease as described in Table 2 and ETNK1 is ethanolamine kinase 1, an enzyme functioning in the first committed step of the phosphatidylethanolamine synthesis pathway. Similarly, the link between BMPER, a protein inhibiting bone morphogenetic and TNFRSF1A (Table 2) would not be an obvious one.

On the contrary, the EPHA5-CFTR pair has some experimental evidence linked to the hypoglycemic response from the ventromedial hypothalamus [21]. They are part of the regulation of neurotransmitter release to adapt the response to varying blood glucose concentrations. Both are already mentioned in Table 2 and Table S1.

Although we found HAVCR2 and CFTR cited together in a 2019 paper related to pancreatic cancer [22], their ratio does not seem biologically meaningful. Likewise, we could not find any link between EPHA5 and HDLBP (Vigilin), which is a protein that plays a role in cell sterol metabolism.

CFTR and KRT16 (Keratin, type I cytoskeletal 16) have been cited together in relation to airway epithelial cells, although the genes were both upregulated along with those of other proteins such as KRT5 in vitro [23]. Other studies focused on various epithelial cells also mention both proteins and their link with tight junctions required for epithelium formation. In our dataset, CFTR is lower in patients (Figure S1) while KRT16 is slightly higher (Supplementary File 1).

Finally, PYCR2 is a Pyrroline-5-carboxylate reductase 2 that catalyzes the last step in proline biosynthesis. Although its primary function in erythrocytes may also be to generate NADP^+^, we were not successful in finding any known link between PYCR2 and Layilin briefly described above.

**Figure 3:**
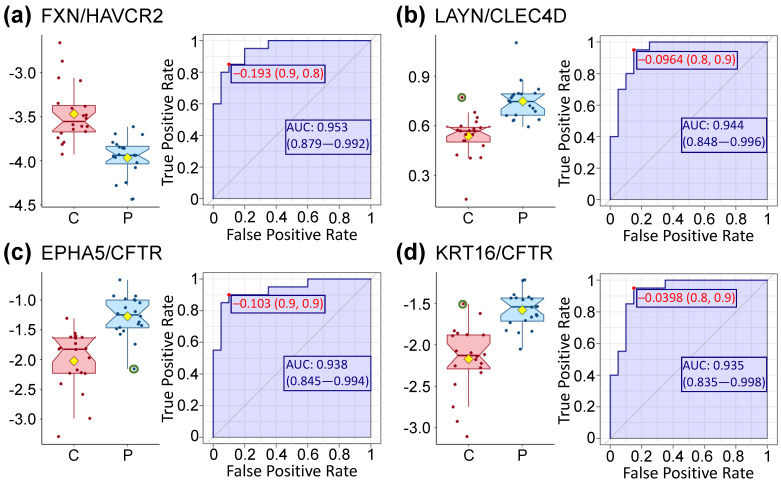
Box plot distribution of the log of the protein ratios displayed in Table 4. Controls (C) are shown in red and ME/CFS patients (P) in blue. The values of the ratios for the three circled green subjects are above (or below) the median and mean of the other cohort. The yellow diamond represents the mean. The corresponding ROC curves are paired with each box plot and include the optimal cutoff (in red) along with the area under the curve (AUC). Protein abundance values are comparable within ratios, with a minimum of ≈200 for FXN and a maximum of ≈3200 for HAVCR2.

As can be seen from this brief description, we were able to provide putative rationales for four of the nine ratios from Table 4. All four ratios are of critical interest as they allow an extremely high level of distinction between controls and patients within our population (AUCs in Figure 3 and Table 4). The detailed values of the four ratios are displayed in Figure 3 where it can easily be seen that most of the values are distinct between cohorts with only three subjects (circled in green) crossing the calculated mean and median of the other cohort. A *t*-test shows that the differences between cohorts for those four ratios are significantly different with *p*-values lower than 0.000003 and even lower using a Wilcoxon test.

### 3.6. Highly Enriched Clusters Include Ephrin-Related Pathways and Glucose

Functional annotation clustering explores the biological meaning behind big datasets such as the one we are manipulating here. The first 3000 UniProt identifiers with the lowest *p*-values were submitted to the DAVID Bioinformatics Resources 6.8 online tool and the analysis was restricted to GeneOntology Direct categories. The results with enrichment scores higher than five are reported in Table 5, and most GO term *q*-values are significantly lower than a stringent threshold of *q* < 0.05.

The second cluster mainly relates to the regulation of the Janus kinase (JAK) activity, which transduces cytokine-mediated signals via the JAK-STAT pathway. The STAT (Signal Transducer and Activator of Transcription) proteins are transcription factors involved in immunity, proliferation, apoptosis, and differentiation.

All three GO terms in the third cluster are biological processes centered around glucose, with GO_061621 being the process that begins conversion of glucose to glucose-6-phosphate; GO_0006096 being reactions and pathways breaking down a carbohydrate into pyruvate; GO_0006094 the reverse reactions that form glucose from noncarbohydrate precursors including pyruvate, amino acids and glycerol.

The fourth cluster revolves around signaling involved in axon guidance as well as cell migration, and function intrinsically with the ephrin-Eph signaling pathway in repulsion or attraction of a cell membrane to a neighboring cell.

Finally, and similarly, the fifth cluster is linked to the ephrin-Eph signaling pathway. The phosphatidylinositol 3-kinases (PI3K) and its downstream signaling are extremely complex and nested in very diverse cellular functions central to cell proliferation as well as neural long-term potentiation.

### 3.7. Protein–Protein Correlations Are Highly Disrupted in the Patient Cohort

We explored the potential protein interaction disruptions between each cohort by calculating each protein–protein interaction within each cohort. We then subtracted the correlation value of ME/CFS patients from controls and sorted the output to find the most contrasting correlations between cohorts. Out of the almost 25 million possible correlations, we selected for the ones that were below −1 (15,505) or above 1 (17,464) for a total of 32,969 disrupted correlations (0.1%).

The lowest value was at −1.66 for the pair C3-ZPBP2 (correlation in controls equals −0.86 and *q* = 0.0003 versus correlation in ME/CFS patients equals 0.8 and *q* = 0.0003). Al-though the Zona pellucida-binding protein 2 is described as being implicated in sperm–oocyte interaction during fertilization, the lower expression of this gene in peripheral blood cells is associated with a reduced risk of asthma in females but not males. In mice, the loss of this protein impacts airway hypersensitivity and lung lipid metabolism in a sex-dependent manner. On the other hand, C3 plays a central role in the activation of the complement system. The complement system is part of the immune system and promotes inflammation to clear microbes and damaged cells. Some of the proteins that are part of this response act as chemoattractant for neutrophils in chronic inflammation, induce the contraction of smooth muscles, increase vascular permeability, and cause histamine release from mast cells and basophilic leukocytes.

The highest value was at 1.54 for the pair NSDHL-IMPAD1 (correlation in controls equals 0.85 and *q* = 0.0005 versus correlation in ME/CFS patients equals −0.69 and *q* = 0.03). Sterol-4-alpha-carboxylate 3-dehydrogenase, decarboxylating (NSDHL) is involved in cholesterol biosynthesis and inositol monophosphatase 3 (IMPAD1) may play a role in the formation of skeletal elements; two visibly different pathways.

**Figure 4:**
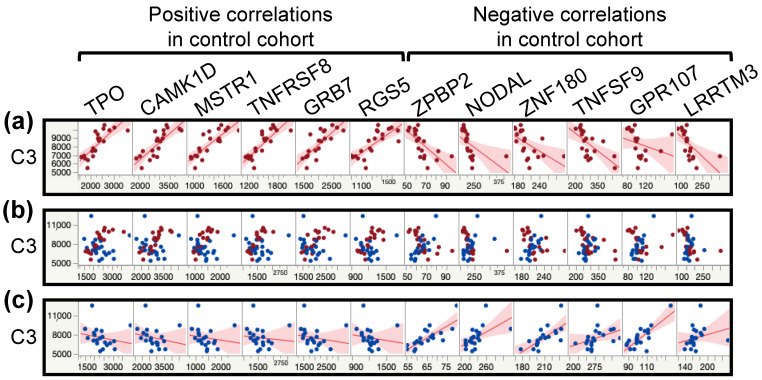
Scatterplot matrices from multivariate analysis between the protein C3 and 12 other proteins with the most drastic correlation changes between the control and the ME/CFS cohorts. Controls (C) are shown in red and ME/CFS patients (P) in blue. (**a**) Scatterplot matrices for the control cohort with fitted line. (**b**) Scatterplot matrices overlay of both cohorts. (**c**) Scatterplot matrices for the ME/CFS cohort with fitted line.

Going through every interaction found to be disrupted in ME/CFS patients compared to controls would distract from the purpose of this analysis. Instead, the principal conclusion is the number of significantly altered interactions that disproportionately affect a few proteins (Table 6). Indeed, CILP2 and CGA FSHB have over 7% of their total interactions inverted in our ME/CFS cohort compared to the control cohort. Moreover, while most interactions are affected by inverting either negative or positive interactions, C3 is a protein that has an equal number of inverted negative and positive correlations. Figure 4 illustrates this point for 12 proteins, six for each kind of correlation, where we can clearly see the flipped correlations between Figure 4a,c. The overlay corroborates the variation between cohorts while exhibiting the value overlaps between cohorts (Figure 4b). These changes demonstrate the extent of the changes in the plasma proteome of ME/CFS patients compared to controls.

Our correlation analysis included the clinical data that was collected, but no significant correlations with protein amounts were detected.

## 4. Discussion

The plasma proteomics dataset investigated here is remarkable for ME/CFS as it allows an extensive probing of protein abundance differences between 20 ME/CFS patients compared to 20 healthy controls. The quantification of plasma proteins whose abundances vary over nine orders of magnitude, achieved by the SOMAmer technology, segregates highly abundant proteins such as albumin, globulins and fibrinogen, which typically account for up to 99% of blood proteins, from the numerous other proteins of lower abundance, which account for another 4773 unique proteins in our dataset. Such a large dataset coupled with an exploratory sized cohort calls for concessions in the analysis and interpretation of results, a balance we have tried to achieve by combining stringent with more relaxed statistical analyses as well as some exploratory tools such as pathway and correlation analyses.

### 4.1. Most Significantly Different Proteins Are More Abundant in the Plasma of ME/CFS Patients

The classic statistical approach, using a Wilcoxon test and multiple testing correction, highlighted nine proteins at a low false discovery rate (FDR) of *q* < 0.05, with one group linked to cellular structure through the cytoskeleton and the extracellular matrix, and a second group linked to the immune system. Of the three proteins related to cellular structure, AIF1L is involved in the cytoskeletal apparatus as an actin-bundling protein [24]. Al-though lower levels of AIF1L have been linked to poor prognosis during breast cancer, AIF1L overexpression in a cell line, similar to what is observed for our ME/CFS cohort (Table 2 and Figure 1), suppressed cell spreading and altered cell shape [25]. The same study predicted a potential role of AIF1L in tight junctions, cell junctions, and focal adhesion, while showing that its overexpression was correlated with decreased RhoA expression, something we also observe in our ME/CFS patient cohort (Figure S2). However, FAK1 (focal adhesion kinase 1) was not concomitantly reduced and instead slightly higher in our patient cohort, short of any further comparison to breast cancer. The human protein atlas (HPA, www.proteinatlas.org) puts most of AIF1L proteins in the kidney and urinary bladder, where it is involved in the stabilization of podocyte morphology and focal adhesions through the actomyosin machinery [26]. AIF1L is also present in many other organs, including male and female reproductive tissues, brain, lungs, the digestive tract, the skin, as well as adipose and soft tissues.

High expression of the extracellular matrix protein PXDN has been linked to proliferation, invasion, and migration of ovarian cancer cell lines, potentially causing an association with poor prognosis in ovarian cancer [27]. PXDN contains a heme-peroxidase domain that allows for its crosslinking activity of collagen IV, a structure crucial to basement membrane synthesis [28]. The basement membrane provides cell and tissue support and acts as a platform for complex signaling. The review article by Peterfi and Geiszt [28] also points to PXDNL, the peroxidasin-like protein homolog to PXDN, exclusively localized in the cell–cell junctions of cardiomyocytes. PXDNL is an antagonist to PXDN when associated in a complex and while our dataset shows higher level of PXDN in the blood of ME/CFS patients, it also displays less PXDNL (*p* = 0.02 and *q* = 0.5, Supplementary File 1). PXDN has been shown to be increased in the heart after cardiac stress or myocardial infarction after activation by elevated levels of TGF-β1 [29].

The third protein, MXRA7, is ubiquitous to many organs including the brain, endocrine tissues, lungs, the pancreas, male and female tissues, as well as muscle tissues and the skin. It is hypothesized that MXRA7 is involved in injury recovery, neovascularization and wound healing [30]. A more recent study shows a high expression of MXRA7 in the basal layer of the human epidermis. The absence of the protein in the mouse model leads to a skin disorder similar to psoriasis, indicating its inhibitory role in skin proliferation [31]. MXRA7 is activated by a few proinflammatory Th1/Th17-type cytokines [32,33]. Other groups have described MXRA7 as necessary to alleviate acute liver injury [34] and a regulator of stem cell differentiation in bone marrow [35].

The four proteins related to the immune system included TIMD3, with high level of protein in bone marrow and lungs, according to HPA; IL-18 BPa, also with high levels in bone marrow; Ephrin-A4 and TNF sR-I seem ubiquitous to all organs. If we focus on the blood atlas from HPA, three of them (TIMD3, TNF sR-I and IL-18 BPa) are secreted into the blood and are present at high levels in almost all immune cells, except TIMD3 and TNF sR-I are not detected in B-cells. In contrast, none of the proteins in the cellular structure group are thought to be regularly secreted into the blood.

TIMD3 is highly expressed in T-cells and myeloid dendritic cells, implicating a role in both innate and adaptive immunity, with loss-of-function mutations leading to autoimmune disorders in 20% of patients, resulting from uncontrolled immunological activation [36]. Additionally noted by Dixon et al. [36] is the high expression of TIMD3 in ‘exhausted’ T-cells in cancers and chronic viral infections, although the associated upregulation of the expression of PD-1 (programmed cell death protein 1) was not observed in our patient cohort (*p* = 0.6 and *q* = 0.9, Supplementary File 1). Nevertheless, high TIMD3 expression inhibits effector T-cell responses and the infiltration of T lymphocytes in adipose tissues, partially increasing the inflammation around adipose tissues in patients [36].

TNF sR-I dysfunction has mainly been described as resulting from mutations disrupting the activity of the protein, leading to the autosomal dominant autoinflammatory syndrome known as TNFR1-associated periodic syndromes (TRAPS) [37]. The underlying mechanism specifically impacts CD4+ conventional T-cells and T_regs_, leading to inflammation [38]. Only membrane bound receptors play a role in cell survival, apoptosis, and inflammation, while its soluble form can capture free tumor necrosis factor alpha (TNF-α), henceforth inhibiting inflammation. The level of TNF ligand is not different between cohorts in our dataset (*p* = 0.8, *q* = 1, Supplementary File 1).

IL-18 BPa prevents the binding of the proinflammatory cytokine IL-18 to its receptor, and in the process reduces both T-helper type 1 and 2 immune responses and overall inflammatory response [39]. IL-18 levels are comparable in our dataset between the control cohort and ME/CFS patients (*p* = 0.8, *q* = 1, Supplementary File 1), which separates this cohort from Crohn’s disease patients, where both IL-18 and IL-18 BPa are higher in patients compared to healthy controls [40]. The increase in IL-18 BPa was not the result of increased IFNγ [41], which is slightly lower in the ME/CFS cohort (*p* = 0.1, *q* = 0.7, Supplementary File 1). IL-18 BPa is strongly upregulated during inflammation, including malignancies such as cancer but also by some viral infections, with some viruses having evolved the capability to express active viral forms of IL-18 BPa [41].

While none of the other proteins highlighted in Table 2 have ever been linked to ME/CFS in plasma, cytokines such as TNF-α and IL-18, and by association the proteins involved in their metabolism, have been the focus of several studies [4,14,42]. Unfortunately, studies of different cohorts of ME/CFS cases and controls from different investigators are divergent in findings concerning cytokine levels.

Understanding the significance of the membrane-bound Ephrin-A4 presents a challenge because it is involved in a myriad of cellular processes. The ephrin receptor signaling pathway will be discussed at greater length below. It is, however, noteworthy that EFNA4, EFNA5 as well as TNFRSF1α were among the upregulated proteins of a correlation network uniquely associated with the regulation of actin filament process, ephrin receptor signaling, and regulation of muscle system processes in a heart failure study [43]. Furthermore, almost all the other proteins of that network were upregulated in our ME/CFS patient cohort (Supplementary File 1).

Finally, even though we did not include RNase1 (Table 2) in either group, it is reported by HPA to be highly expressed in dendritic cells and classic monocytes as well as secreted to the digestive system. Of the remaining proteins present in Tables S1 and S2, DCP1A is the only one highly expressed in all immune cells, while DR6 is specific to dendritic cells.

CFTR is another gene that currently has a modest link with ME/CFS when gene-expression data was used in an attempt to select potential FDA-approved drugs that can be repurposed as putative treatments for ME/CFS [44]. CFTR is part of the identified gene module “M46-recycling pathway of L1”, linked to the drug Ivacaftor, which is used to increase the ion-function of the activated cell-surface CFTR channel in individual homozygous for a particular CFTR mutation [45]. Ivacaftor therapy changes blood monocyte transcriptional profiles and plasma chemokines in patients with cystic fibrosis [46]. Additionally, CFTR was highlighted in two of the four ratios (Figure 3c,d) we selected as having some biological significance. As displayed in Figure S1g, CFTR is one of the few proteins we are discussing that is lower in our ME/CFS patients cohort compared to healthy controls. CFTR is a member of the ATP-binding cassette (ABC) transporter superfamily and functions as a chloride and bicarbonate ion channel. Individuals heterozygous for CFTR mutations, who have reduced CFTR activity, are at greater risk for a number of disorders common in cystic fibrosis patients [47]. CFTR regulates many mechanisms in epithelial physiology, such as maintaining epithelial surface hydration and regulating luminal pH [48]. As summarized by Saint-Criq and Gray [48], CFTR plays a fundamental role in regulating secretion and absorption throughout the body, including airways, the gastrointestinal and reproductive tracts, sweat and salivary glands. Additionally, its role in luminal pH is an important arbiter of epithelial barrier function and innate defense [48]. The KRT16 and CFTR proteins have been cited together and several studies linked to airway-specific markers [23,49]. KRT16 is part of the keratin family, filament proteins responsible for the structural integrity of epithelial cells and is present in bone marrow, skin, lungs, and the proximal digestive tract according to HPA. Moreover, KRT16, along with several other proteins from the same large family, has already been found to be significantly different in the cerebrospinal fluid of a primarily male ME/CFS cohort compared to healthy controls [50]. The EPHA5 and CFTR proteins are both implicated as functioning in the ventromedial hypothalamus (VMH) at the level of synaptic neuron-glia in response to hypoglycemia by enhancing glutamatergic neurotransmission [21]. There are drugs either available or being developed for cystic fibrosis; ones that might improve CFTR function in individuals with reduced levels may be worth further investigation in ME/CFS patients whose levels are low.

Overall, protein ratios enhance the distinction between ME/CFS patients and controls (AUC = 0.95) compared to individual proteins, for which we obtained similar values as previously reported in various studies [9,11].

### 4.2. Much Evidence Implicates Disrupted Ephrin-Eph Signaling in Our Dataset

The Eph proteins from the superfamily of transmembrane tyrosine kinase receptors as well as their membrane-tethered ephrin ligands appear, directly or indirectly, in many of our figures and tables (Figure 1, Figure 2 and Figure 3 and Figure S2 and Table 2, Table 3, Table 4 and Table 5, Tables S2 and S3). This protein family promotes cell–cell signaling and coordinates a myriad of developmental processes including neural map ordering. Its importance extends into adulthood where family members regulate neuronal plasticity, homeostatic events, and disease processes [51]. Kania and Klein [51] also describe how ephrin-Eph signaling (1) modulates the cytoskeleton through RhoA (shown in Figure 2 and Figure S2), (2) affects neural development through either repulsion or adhesion leading to axon repulsion, synapse formation, axon tract formation, axon pruning, cell migration, with obvious considerable consequences, (3) functions in tissue separation in all organs, including the brain and blood vessels to name a few. The proteome of ME/CFS patients’ cerebrospinal fluid has led to conclusions centered around axonal guidance pathways, also linked to the ephrin-Eph signaling pathway, along with an interest in the complement pathways, with C3 being one of them, which has a protein correlation network that is severely disrupted in our dataset (Table 6) [52].

It is recognized that ephrin-Eph signaling cascades are redeployed in adults to control the cytoskeleton and therefore cellular morphology, cell–cell signaling, stem cell niche maintenance, neuronal synaptic stability as well as energy metabolism [51]. The latter is derived from pancreatic islet β-cells and the regulation of insulin secretion, controlling a fundamental process underlying energy metabolism. Indeed, the Human Protein Atlas reported CFTR as being highly expressed in the pancreas and the liver. Finally, Kania and Klein [51] conclude with links between Eph-ephrin signaling and disease, with a robust decade of research connecting the pathway to tumor growth, pathological angiogenesis, and malignant cell migration, but also to neurological disorders such as lateral sclerosis or Alzheimer’s disease. The connection with neurological problems may be significant in the context of ME/CFS due to the common symptom of cognitive dysfunction.

Other substantial roles of the ephrin-Eph signaling pathway lie in immunity as outlined throughout an extensive review covering stem cell fate, immune cell activation, immune cell trafficking, and again some disease pathogenesis such as cancer, atherosclerosis, fibrosis, diseases of the central nervous system, and infectious diseases [53]. A more recent review details how genetically diverse viruses utilize Eph receptors for viral entry [54], including Epstein–Barr virus, which has a controversial association with ME/CFS [55,56]. Nevertheless, a recent study focused on circulating extracellular vesicles in ME/CFS patients finds evidence for many aspects in which the ephrin-Eph pathway is involved, including PI3K, mentioned during our cluster analysis, actin skeletal regulation and focal adhesions [57].

The concurring evidence from the analysis of our dataset highlights the ephrin-Eph signaling pathway as a central component disrupted in our 20 ME/CFS patients compared to the 20 healthy controls. When we review the extensive literature on an extensive superfamily of ligand and receptors, it is easy to link many of the symptoms endured by patients to the numerous processes controlled through the complex ephrin-Eph signaling pathway. As featured in Figure 2, all of the proteins gravitating around RhoA on the ephrin side are part of the ephrin-A class, which are anchored to the membrane by a glycosylphosphatidylinositol linkage, whereas those in the ephrin-B class are transmembrane proteins. Nevertheless, there is some cross-recognition between classes with ephrin-B3 able to bind to EphrA4 and ephrin-A5 to EphB2 [58]. The other group of proteins in Figure 2 are all related to myosin regulation in both smooth muscle and non-muscle cell contraction, cell polarity, actin polymerization, focal adhesions, and motility, according to the HPA. If the association of this pathway with ME/CFS is replicated in a larger cohort, this pathway may become a target for experimental therapy for ME/CFS. Agents inhibiting the usage of the ephrin-Eph pathway for viral entry are known, but have been studied only in vitro as yet [54].

The plasma origin of our dataset must be kept in mind when interpreting protein differences and especially pathway analysis, specifically when primarily known to be resident in specific tissues and organs. Nevertheless, proteins from a variety of tissues can be detected in plasma and have been able to serve as biomarkers, either resulting from secretion, cell damage or senescence. Furthermore, proteins that may be secreted from circulating immune cells can provide insight into the functioning of the immune system in the two cohorts. To our knowledge, an association with the ephrin-Eph pathway has not previously been reported in ME/CFS, highlighting the importance of methods that allow analysis of many proteins exhibiting wide variations in concentration.

## 5. Conclusions

We used assorted tools to explore the largest proteomics dataset in ME/CFS to date, spanning nine orders of magnitude of plasma protein abundance. While our cohort was small in this exploratory study, we nevertheless were able to detect proteins with levels that were either significantly different or strongly affected in the patient cohort compared to the controls. We identified nine proteins with individual classifiers greater than 0.85 between ME/CFS subjects and controls as well as nine protein ratios with classifiers above 0.92. As is practice, a diagnostic test for ME/CFS would not be used on subjects who are not complaining of fatigue or malaise; these protein differences must be tested against other fatiguing illness that might be confused with ME/CFS—such as depression, cancer, or chronic Lyme disease, to name a few. Such studies will be needed to determine how the sensitivity and specificity are affected by excluding individuals who do not exhibit fatigue or malaise.

Overall, these encouraging results illustrate the power of large-scale studies to investigate a disease whose molecular basis still remains opaque.

## 6. Patents

A provisional patent application (U.S. Serial No. 63132722) concerning the data has been filed by Cornell University.

## Figures and Tables

**Table 1:** Details of the population statistics.

Gender (*n*)	Female	Controls	ME/CFS
		20	20
**Age**	Mean +/− SD	46 +/− 13.3	49.2 +/− 11.8
	Median (min-max)	50.5 (27–66)	52 (27–68)
**BMI**	Mean +/− SD	23.2 +/− 3.4	24 +/− 5.1
	Median (min-max)	21.8 (18.1–29.3)	22.6 (16.8–37.4)
**Type of onset**	Gradual	NA	35%
	Sudden	NA	65%
Gut symptoms *		5%	55%
Positive tilt table test ** (*n* = 12)		ND	67%
Bell’s disability scale ***	10–20	0	7
	30–40	0	7
	50–60	1	6
	90–100	19	0
SF-36 ***	Physical component summary (PCS)	55.4 +/− 5.9	27 +/− 8.6
	Mental component summary (MCS)	53.6 +/− 9.6	37.6 +/− 11.8

* Subjects reporting either irritable bowel syndrome (IBS), ulcerative colitis, or Crohn’s disease. ** A tilt test had previously been performed on 12 of the ME/CFS patients, and eight were positive. *** Higher scores represent better health. NA: not applicable. ND: not determined.

**Table 2:** Proteins significantly different between controls and patients through Wilcoxon rank-sum testing with *p* < 0.05 and *q* < 0.05 of Supplementary File 1.

Protein	Full Name	UniProt	EntrezGene	Fold Change	*p*-Value	*q*-Value
**RNase1**	Ribonuclease pancreatic	P07998	RNASE1 (6035)	1.47	0.00002	0.035
**RABP2**	Cellular retinoic acid-binding protein 2	P29373	CRABP2 (1382)	1.47	0.00002	0.035
**AIF1L**	Allograft inflammatory factor 1-like	Q9BQI0	AIFL1 (83543)	1.25	0.00002	0.035
**PXDN**	Peroxidasin homolog	Q92626	PXDN (7837)	1.39	0.00005	0.038
**Ephrin-A4**	Ephrin-A4	P52798	EFNA4 (1945)	1.16	0.00005	0.038
**TIMD3**	Hepatitis A virus cellular receptor 2	Q8TDQ0	HAVCR2 (84868)	1.1	0.00006	0.038
**MXRA7**	Matrix-remodeling-associated protein 7	P84157	MXRA7 (439921)	1.14	0.00006	0.038
**TNF sR-I**	Tumor necrosis factor receptor superfamily member 1A	P19438	TNFRSF1A (7132)	1.28	0.00007	0.038
**IL-18 BPa**	Interleukin-18-binding protein	O95998	IL18BP (10068)	1.24	0.00007	0.038

Fold change represents the ratio from group means of patients/controls.

**Table 3:** STRING output of detected functional enrichments resulting from the submission of the complete dataset along with the associated log *p*-value for each protein.

Term ID *	Term Description	Enrichment Score	Genes Mapped	Pathway Size	False Discovery (afc)
HSA-3928663	EPHA-mediated growth cone collapse	2.26367	17	28	0.00051
CL:416	Ephrin receptor activity, and Ephrin	2.07704	20	23	0.00038
CL:420	Ephrin receptor activity, and Ephrin	2.72701	13	16	0.00038
IPR031328	Ephrin	3.36604	7	9	0.0049
IPR019765	Ephrin, conserved site	3.36604	7	9	0.0049
IPR001799	Ephrin receptor-binding domain	3.36604	7	9	0.0049
IPR034252	Ephrin-A ectodomain	5.24701	4	6	0.0049

* Term IDs come from several functional enrichments. CL refers to local STRING network cluster; HSA to Reactome Pathways; IPR to INTERPRO Protein Domains and Features. An additional 37 “Reference publications” are omitted but can be easily retrieved by interested parties using data from Supplementary File 1, namely, columns “EntrezGeneSymbol” and the logged values of column “*p*-value”.

**Table 4:** Proteins significantly different between controls and patients through Wilcoxon rank-sum testing with *p* < 0.05 and *q* < 0.05 of Supplementary File 1.

Protein	AUC	Protein Ratio	EntrezGene	UniProt	AUC
**RNase1**	0.87	FRDA/TIMD3 *	FXN/HAVCR2 *	Q16595/Q8TDQ0	0.95
**RABP2**	0.87	Layilin/CLC4D *	LAYN/CLEC4D *	Q6UX15/Q8WXI8	0.94
**AIF1L**	0.87	**RNase1/EKI1**	RNASE1/ETNK1	P07998/Q9HBU6	0.94
**PXDN**	0.86	**BMPER/TNF sR-I**	BMPER/TNFRSF1A	Q8N8U9/P19438	0.93
**Ephrin-A4**	0.86	EphA5/CFTR *	EPHA5/CFTR *	P54756/P13569	0.93
**TIMD3**	0.85	**TIMD3/CFTR**	HAVCR2/CFTR	Q8TDQ0/P13569	0.93
**MXRA7**	0.85	**VIGLN/EphA5**	HDLBP/EPHA5	Q00341/P54756	0.93
**TNF sR-I**	0.85	Keratin-16/CFTR *	KRT16/CFTR *	P08779/P13569	0.93
**IL-18 BPa**	0.85	**P5CR2/Layilin**	PYCR2/LAYN	Q96C36/Q6UX15	0.92

* Ratios with potential biological relevance.

**Table 5:** Details of the functional annotation clustering results with an enrichment score above five.

Annotation Cluster	Enrichment Score	GO Term		Protein Count	*p*-Value	*q*-Value
1	14.3	CC_GO:0005913	Cell–cell adherens junction	113	4.46 × 10^−18^	6.72 × 10^−16^
		MF_GO:0098641	Cadherin binding involved in cell-cell adhesion	98	1.59 × 10^−14^	3.49 × 10^−12^
		BP_GO:0098609	Cell–cell adhesion	90	2.46 × 10^−12^	9.64 × 10^−10^
2	6.5	BP_GO:0038083	Peptidyl-tyrosine autophosphorylation	24	2.06 × 10^−9^	3.93 × 10^−7^
		MF_GO:0004715	Nonmembrane spanning protein tyrosine kinase activity	22	1.21 × 10^−6^	0.00009
		CC_GO:0031234	Extrinsic component of cytoplasmic side of plasma membrane	26	0.00001	0.0004
3	5.5	BP_GO:0061621	Canonical glycolysis	17	1.65 × 10^−7^	0.00002
		BP_GO:0006096	Glycolytic process	18	3.70 × 10^−6^	0.0003
		BP_GO:0006094	Gluconeogenesis	19	0.00006	0.003
4	5.1	BP_GO:0050919	Negative chemotaxis	21	1.37 × 10^−8^	2.17 × 10^−6^
		MF_GO:0045499	Chemorepellent activity	18	2.85 × 10^−8^	2.81 × 10^−6^
		BP_GO:0048843	Negative regulation of axon extension involved in axon guidance	15	8.89 × 10^−6^	0.0007
		MF_GO:0030215	Semaphorin receptor binding	13	0.00005	0.002
		BP_GO:0071526	Semaphorin-plexin signaling pathway	16	0.00006	0.003
		MF_GO:0038191	Neuropilin binding	10	0.0001	0.004
		BP_GO:0001755	Neural crest cell migration	16	0.003	0.07
		BP_GO:0008543	Fibroblast growth factor receptor signaling pathway	34	9.67 × 10^−8^	0.00001
5	5	BP_GO:0048015	Phosphatidylinositol-mediated signaling	40	1.22 × 10^−7^	0.00002
		MF_GO:0046934	Phosphatidylinositol-4,5-bisphosphate 3-kinase activity	28	1.26 × 10^−7^	0.00001
		MF_GO:0005088	Ras guanyl-nucleotide exchange factor activity	41	2.52 × 10^−7^	0.00002
		BP_GO:0014066	Regulation of phosphatidylinositol 3-kinase signaling	32	3.27 × 10^−7^	0.00004
		BP_GO:0046854	Phosphatidylinositol phosphorylation	32	0.00003	0.002
		MF_GO:0005104	Fibroblast growth factor receptor binding	12	0.0002	0.006
		MF_GO:0016303	1-phosphatidylinositol-3-kinase activity	13	0.03	0.35
		BP_GO:0036092	Phosphatidylinositol-3-phosphate biosynthetic process	14	0.04	0.46

Within GO term, CC, MF, and BP stand for cellular component, molecular function, and biological process, respectively.

**Table 6:** Proteins with inverted correlations between the control and the ME/CFS cohorts.

Protein	Negative Correlations in Controls Positive Correlations in ME/CFS	Protein	Positive Correlations in Controls Negative Correlations in ME/CFS
**CGA FSHB**	347 (349)	**CILP2**	381 (386)
**FIS1**	281 (340)	**CASC4**	301 (308)
**TNXB**	251 (331)	**TMEM9**	285 (287)
**CGA LHB**	226 (230)	**ENO3**	280 (296)
**RNF215**	211 (242)	**VWA2**	267 (291)
**RCN3**	209 (306)	**AMN**	222 (240)
**RNF215.1**	202 (242)	**CLIC5**	200 (205)
**C3**	195 (359)	**CACNA2D3**	193 (200)
**GRB14**	194 (218)	**OBP2B**	183 (190)
**FIGF**	144 (271)	**THSD7A**	174 (231)
**BRD2**	129 (132)	**C3**	164 (359)
**ANTXR1**	124 (166)	**TTC9B**	157 (257)
**C1orf210**	105 (182)	**LAMC2**	143 (151)
**IGFBP1**	101 (134)	**PIANP**	129 (133)
**TTC9B**	100 (257)	**TRAPPC3**	129 (181)

Numbers in parentheses are the total from both types of inverted correlations.

## Data Availability

The data supporting the reported results can be found at https://www.mapmecfs.org.

## Supplementary Materials

The following are available online at https://www.mdpi.com/2227-7382/9/1/6/s1, Figure S1: Box plot distribution of logged values for the proteins significantly different between controls and patients through Wilcoxon rank-sum testing with *p* < 0.05 and 0.05 < *q* < 0.15 of Supplementary File 1, also displayed in Table 3. Controls (C) are shown in red and ME/CFS patients (P) in blue. The yellow diamond represents the mean. * BAMBI was “flagged” based on SomaLogic’s acceptance criteria. Figure S2: Box plot distribution of logged values for the proteins associated with HSA-3928663 from Table 3 and Table S3 with *p* < 0.15. Controls (C) are shown in red and ME/CFS patients (P) in blue. The yellow diamond represents the mean. Table S1: List of proteins significantly different between controls and patients through Wilcoxon rank-sum testing with *p* < 0.05 and 0.05 < *q* < 0.15 of Supplementary File 1. Table S2: List of proteins significantly different between controls and patients through Wilcoxon rank-sum testing with *p* < 0.05 and *q* < 0.2. Table S3: List of proteins associated with HSA-3928663 from Table 3 with *p* < 0.15. Supplementary File 1: List of proteins with *p*-values, *q*-values, fold changes of patients/controls (FC) and log2(FC). Supplementary File 2: List of AUC values from protein ratios and single proteins above 0.75.
