# Supplementary material for: In-Depth Analysis of the Plasma Proteome in ME/CFS Exposes Disrupted Ephrin-Eph and Immune System Signaling
Source: Proteomes. 2021 Jan 29;9(1):6. doi: 10.3390/proteomes9010006 (PMC7931008; doi:10.3390/proteomes9010006)
Supplement: Supplementary file 1 [file proteomes-09-00006-s001.zip › Germain_Supplemental.docx]

Supplemental Materials

In-depth analysis of the plasma proteome in ME/CFS exposes disrupted ephrin-Eph and immune system signaling

Arnaud Germain, Susan M. Levine and Maureen R. Hanson*

**Figure S1.** Box plot distribution of logged values for the proteins significantly different between controls and patients through Wilcoxon rank-sum testing with *p* < 0.05 and 0.05 < *q* < 0.15 of Supplementary File 1, also displayed in Table 3. Controls (C) are shown in red and ME/CFS patients (P) in blue. The yellow diamond represents the mean.

* BAMBI was “flagged” based on SomaLogic’s acceptance criteria.

**Figure S2.** Box plot distribution of logged values for the proteins associated with HSA-3928663 from Tables 3 and S3 with *p* < 0.15. Controls (C) are shown in red and ME/CFS patients (P) in blue. The yellow diamond represents the mean.

**Table S1.** List of proteins significantly different between controls and patients through Wilcoxon rank-sum testing with *p* < 0.05 and 0.05 < *q* < 0.15 of Supplementary File 1.

| **Protein** | **Full Name** | **UniProt** | **EntrezGene** | **Fold Change** | ***p*-value** | ***q*-value** |
| --- | --- | --- | --- | --- | --- | --- |
| **ROR1** | Inactive tyrosine-protein kinase transmembrane receptor ROR1 | Q01973 | ROR1 (4919) | 1.3 | 0.0002 | 0.08 |
| **DCP1A** | mRNA-decapping enzyme 1A | Q9NPI6 | DCP1A (ND) | 1.3 | 0.0002 | 0.08 |
| **TPPP2** | Tubulin polymerization-promoting protein family member 2 | P59282 | TPPP2 (ND) | 1.4 | 0.0002 | 0.09 |
| **F159A** | Membrane protein FAM159A | Q6UWV7 | FAM159A (ND) | 1.3 | 0.0002 | 0.09 |
| **PPIC** | Peptidyl-propyl cis-trans  isomerase C | P45877 | PPIC (5480) | 1.2 | 0.0003 | 0.12 |
| **DAN** | Neuroblastoma suppressor of tumorigenicity 1 | P41271 | NBL1 (4681) | 1.2 | 0.0004 | 0.12 |
| **CFTR** | Cystic fibrosis transmembrane conductance regulator | P13569 | CFTR (1080) | 0.7 | 0.0004 | 0.12 |
| **BAMBI*** | BMP and activin membrane-bound inhibitor homolog | Q13145 | BAMBI (25805) | 1.4 | 0.0004 | 0.14 |

Fold change represents the ratio from group means of patients/controls.

* BAMBI was “flagged” based on SomaLogic’s acceptance criteria.

**Table S2.** List of proteins significantly different between controls and patients through Wilcoxon rank-sum testing with *p* < 0.05 and *q* < 0.2.

| **Protein** | **Full Name** | **UniProt** | **EntrezGene** | **Fold Change** | | ***p*-value** | ***q*-value** |
| --- | --- | --- | --- | --- | --- | --- | --- |
| **DR6** | Tumor necrosis factor receptor superfamily member 21 | O75509 | TNFRSF21 (27242) | 1.1 | | 0.00002 | 0.11 |
| **PPIC** | Peptidyl-propyl cis-trans  isomerase C | P45877 | PPIC (5480) | 1.2 | | 0.0002 | 0.19 |
| **ROR1** | Inactive tyrosine-protein kinase transmembrane receptor ROR1 | Q01973 | ROR1 (4919) | | 1.3 | 0.0002 | 0.19 |
| **Ephrin-A5** | Ephrin-A5 | P52803 | EFNA5 (1946) | 1.2 | | 0.0008 | 0.19 |

Fold change represents the ratio from group means of patients/controls.

**Table S3.** List of proteins associated with HSA-3928663 from Table 3 with *p* < 0.15.

| **Protein** | **Full Name** | **UniProt** | **EntrezGene** | **Fold Change** | ***p*-value** | ***q*-value** |
| --- | --- | --- | --- | --- | --- | --- |
| **Ephrin-A4** | Ephrin-A4 | P52798 | EFNA4 (1945) | 1.16 | 0.00005 | 0.038 |
| **Ephrin-A5** | Ephrin-A5 | P52803 | EFNA5 (1946) | 1.23 | 0.0008 | 0.19 |
| **Ephrin-A2** | Ephrin-A2 | O43921 | EFNA2 (1943) | 1.17 | 0.0009 | 0.19 |
| **EphA5** | Ephrin type-A receptor 5 | P54756 | EPHA5 (2044) | 1.23 | 0.003 | 0.31 |
| **EphA1** | Ephrin type-A receptor 1 | P21709 | EPHA1 (2041) | 1.25 | 0.006 | 0.37 |
| **FYN** | Tyrosine-protein kinase Fyn | P06241 | FYN (2534) | 0.69 | 0.01 | 0.43 |
| **EphA2** | Ephrin type-A receptor 2 | P29317 | EPHA2 (1969) | 1.1 | 0.04 | 0.62 |
| **Rho A** | Transforming protein RhoA | P61586 | RHOA (387) | 0.8 | 0.05 | 0.64 |
| **LYN** | Tyrosine-protein kinase Lyn | P07948 | LYN (4067) | 0.8 | 0.06 | 0.65 |
| **EphA7** | Ephrin type-A receptor 7 | Q15375 | EPHA7 (2045) | 1.15 | 0.11 | 0.71 |

Fold change represents the ratio from group means of patients/controls.
